# Using Business Analytics for SME Business Model Transformation under Pandemic Time Pressure

**DOI:** 10.1007/s10796-022-10255-8

**Published:** 2022-03-02

**Authors:** Efpraxia D. Zamani, Anastasia Griva, Kieran Conboy

**Affiliations:** 1 Information School, The University of Sheffield, Sheffield, UK; 2 Lero – The Science Foundation Ireland Research Centre for Software, School of Business & Economics, National University of Ireland Galway, Galway, Ireland

**Keywords:** Busines analytics, Busines model innovation, Business model adaptation, Exogenous shock, SMEs, Dynamic capabilities

## Abstract

The COVID-19 pandemic has had an unprecedented impact on many industry sectors, forcing many companies and particularly Small Medium Enterprises (SMEs) to fundamentally change their business models under extreme time pressure. While there are claims that technologies such as analytics can help such rapid transitions, little empirical research exists that shows if or how Business Analytics (BA) supports the adaptation or innovation of SMEs’ business models, let alone within the context of extreme time pressure and turbulence. This study addresses this gap through an exemplar case, where the SME actively used location-based business analytics for rapid business model adaptation and innovation during the Covid-19 crisis. The paper contributes to existing theory by providing a set of propositions, an agenda for future research and a guide for SMEs to assess and implement their own use of analytics for business model transformation.

## Introduction

The COVID-19 pandemic has forced many organisations to undergo significant transformation, rethinking key elements of their business models and use of technology to maintain operations whilst adhering to a changing landscape of guidelines and new procedures (Dwivedi et al., 2020). It has required some businesses not just to adapt slightly, but instead adopt a completely new perspective on how they deal with change, given the time pressure and ‘liquid’ nature of the environment (Carroll & Conboy, 2020). When businesses find themselves under existential threat, transforming the business model may allow them to reinvent their products and processes and essentially breathe new life into the business (Calia et al., 2007; Kraus et al., 2020). Such changes are known as business model dynamics (Saebi et al., 2017).

While the pandemic has of course affected all industries and all organisations in some way, SMEs have been acutely affected for a number of reasons. First, unlike larger organisations, they are unlikely to have large scale financial reserves for support during transition. Second, SMEs are usually limited in the amount of borrowing they can obtain and also limited in the duration of that borrowing. Third, while large organisations can obtain government support, SMEs are often “left behind” by central government support (Giones et al., 2020, p. 2). Also, given that their limited monthly recuring revenues (MRR) usually derive from a single product, attached to a specific business model, there is limited flexibility to diversify, and they are massively exposed in terms of risk. Fourth, and most relevant in the context of this study, is that while large organisations can utilise emerging technology by investing heavily in expertise, SMEs typically do not have a dedicated data science team; while in practice they do exploit BA to unveil opportunities, they usually do so in an unstructured way (Dereli et al., 2020).

However, Business Analytics (BA) can play a significant role in offering business continuity in times of crisis (Papadopoulos et al., 2020). BA has been identified as holding great potential for future proofing business decisions, as well as resource and capacity planning (Sheng et al., 2020; van Rijmenam et al., 2019). Contemporary digital SMEs do not have many options to innovate and transform their business model, other than leveraging technologies; it is thus essential to exploit digital technologies such as business analytics to support their business model transition (Griva et al., 2021b). Instagram is a well-known example that used BA to alter their business model, whereby during the early-stages, its founders used BA to analyse app data and spot users’ preferences regarding posting photographs (Steer, 2021). However, research-wise, although there is evidence that smaller firms use BA (e.g., Behl et al., 2019; Berg et al., 2018; Sayyed-Alikhani et al., 2021), there is a paucity of research on how exactly SMEs use this technology for business model transformations.

As such, this study aims to understand “how SMEs exploit Business Analytics to change their Business Model during exogenous shocks?” To address our research question, we adopt the theoretical lens of Dynamic Capabilities (DC) to assist us in structuring our empirical observations of the BA-enabled processes that SMEs conduct to ‘sense’ and ‘seize’ emerging opportunities, and consequently ‘transform’ themselves to respond to the new and dynamic environment (Teece, 2007). We note that in the existing literature, although BA-enabled DC have been studied in relation to firm performance (e.g., Fosso Wamba et al., 2017; Torres et al., 2018) and innovation capability (Mikalef et al., 2019), the use of BA-enabled DC for business model dynamics remains largely underexplored (Ciampi et al., 2021).

To address this gap, we present a case study of a start-up called TrackApp. This has been purposefully selected as an exemplar case since the team actively used business analytics for rapid business model adaptation and innovation during the pressure of the COVID-19 crisis. In a nutshell, our findings indicate that the start-up transformed its business model three times. During the first one, they used BA to analyse internal (e.g., financial, app, historical) and external (e.g., social media) datasets, with a view to understand the new environment, and develop a new BA-enabled app feature (i.e., a queue monitoring system for retail stores). In the second transformation, BA served as an enabler for more radical alterations in the Business Model. Using mainly internal datasets, the start-up created a new BA-enabled product that exploits advanced machine learning techniques to track users in indoor retail environments. In the last transformation, BA was used to re-assess the situation and expand the BA capabilities of the new product via adding a recommendation feature.

The paper is structured as follows. The next section presents the background literature on (i) business model dynamics in times of crises, (ii) the use of BA in business model dynamics, (iii) the role of DC in BA, and (iv) the SME context in regard to the above. We then present our research methods, which is followed by a description of the case and the main findings of the study. These findings are discussed in the context of existing literature, in order to highlight the contributions of the study, and to identify directions for future research and limitations of the study.

## Theoretical Background

### Business Models during Times of Crises

The Covid-19 outbreak can be seen as an exogenous shock, just like “large-scale events that impose crises” and lead to an unprecedented need for “radical transformation” (Corbo et al., 2015, pp. 323–324). For organisations, such shocks may indicate the need for significant changes in how value is created, delivered and captured (Breier et al., 2021), i.e., changes in the business model.

Existing literature refers to such changes as business model dynamics, whereby a firm evolves, modifies, reconfigures, innovates or renews its business model, some of which are used interchangeably (Foss & Saebi, 2018). Referring to business model dynamics indicates that there are interactions happening within and across the components of a business model over time and signifies a point of departure from the more static views, which to date are focussed on identifying the configurations of said components rather than how these evolve over time (Demil & Lecocq, 2010). Along these lines, the literature characterises business model dynamics either as business model adaptation (BMA) or as Business Model Innovation (BMI) to differentiate between incremental and radical changes (Foss & Saebi, 2018). Specifically, the differences between BMA and BMI is usually based on the different underlying conditions and motivation of the business model dynamics, and the end result, i.e., the new business model is characterised by a different degree of novelty (Saebi et al., 2017).

BMA comprises all types of business model transformation (e.g., renewal, reconfiguration, evolution) and is defined as “the process by which management actively aligns the firm’s business model to a changing environment, for example, changes in the preferences of customers, supplier bargaining power, technological changes, competition, etc.” (Saebi et al., 2017, p. 569).

BMI is defined as the “designed, novel, nontrivial changes to the key elements of a firm’s business model and/or the architecture linking these elements” (Foss & Saebi, 2017, p. 201). Contrasting BMA and BMI, one can argue that the underlying difference between the two lies with management’s motivation: while BMA seeks to respond to changes (typically in the external environment), BMI seeks to disrupt the status quo (Markides, 2006), and can be potentially the result of BMA (Saebi et al., 2017). For example, during crises, adapting the business model may be the only option to ensure survival (Kraus et al., 2020; Poisson-de Haro & Montpetit, 2012). However, BMI can support businesses to leverage or exploit emerging technologies, as for example the Internet of Things, to challenge and leapfrog their competition, and disrupt mature markets (Haaker et al., 2021).

Along these lines, digital technologies are found at the core of BMs (Pateli & Giaglis, 2005), whereby emerging and disruptive technologies support firms to design, adapt and innovate their BMs (Haaker et al., 2021). This is discussed next, where we concentrate specifically on the use of Business Analytics (BA) for business model dynamics, which is the focus of this study.

### Business Analytics and Business Models Dynamics

Information Technologies (IT) have long been viewed as holding a critical role for a business, whereby an IT strategy was necessary and required to be in alignment with the business strategy. With the increased emphasis on digitalisation and disruptive technologies, however, the focus has changed; from being merely aligned, and thus subordinate, the discourse has now shifted to a digital business strategy being of equal importance to the business strategy (Bharadwaj et al., 2013).

Among these technologies, Business Analytics (BA) is often cited as one with particular potential, given its inherent ability to augment business processes, and functions as a sensitising device (Bygstad et al., 2020; Zamani et al., 2021) that enables the prediction of outcomes and the magnitude of impacts on the firm (Fosso Wamba et al., 2020). For example, BA can support a firm with better informed and rationalised decision-making (Ciampi et al., 2021), by helping decision makers forecast profitability and identify new markets, products and services (Akhtar et al., 2019). Similarly, retailers and manufacturers can predict more accurately market demand, customers’ preferences and requirements (Griva et al., 2021a), and therefore streamline supply chains (Gupta et al., 2020). Equally BA may predict potential risks (Kiron et al., 2014). Therefore, in light of exogenous shocks, BA may indicate more rapidly the more critical business functions, and whether and how these need to adapt to the changing conditions (Sheng et al., 2020).

As far as business models are concerned, in light of an exogenous shock, we argue that BA can highlight new, potentially previously unexplored, market segments. It can thus support crafting a value proposition that targets a particular segment through and on the basis of data (van Rijmenam et al., 2019), whereby BA can indicate ‘responses’ (Bygstad et al., 2020), and such ‘responses’ may entail repositioning the firm against competitive forces or for outlasting the shock. Considering adaptations and innovations of business models, BA can be a lens through which a firm can identify, assess, and determine the viability of alternative scenarios and ultimately identify its pathway to recovery. Namely, BA-inspired adaptations in the targeted market segment or the value proposition may as well lead to adaptations in the value capture mechanisms and the value chain itself (Teece, 2010). Equally, BA-driven adaptations may lead to BMI, too, if the resulting business model allows the firm to disrupt the market and identify markets ignored by the competition, especially if this happens in a manner that is difficult to be imitated by others (Foss & Saebi, 2017).

### Dynamic Capabilities and Business Analytics

Dynamic capabilities are a firm’s abilities “to integrate, build, and reconfigure internal and external competences to address rapidly changing environments” (Teece et al., 1997, p. 516). Dynamic capabilities allow firms to sense and seize emergent business opportunities against the backdrop of a continuously changing environment, and subsequently transform the way they operate with the view to adapt to the newly created market reality (Teece, 2007).

*Sensing* entails being able to identify and assess opportunities within an ever-changing environment. This often happens through the use of analytical systems, for the purposes of scanning the horizon and pinpointing opportunities, or threats (Helfat & Raubitschek, 2018). A firm may adopt search and exploration activities in order to gather market intelligence across markets, often by leveraging innovative technologies (Conboy et al., 2020). Crucially, the firm will need to filter and reduce the amount of information that needs to be interpreted and used in the next step (Torres et al., 2018).

*Seizing* is focussed around the mobilisation of resources in order to address the identified opportunities (Fosso Wamba et al., 2017; Helfat & Raubitschek, 2018) and has at its core the reformulation of the business model (Teece, 2018). This is usually done by evaluating first any existing and emerging capabilities, achieving consensus, which will help the firm overcome inertia and rigidity (Torres et al., 2018) and making the appropriate investments in those areas that seem more viable, i.e., where market acceptance seems likely (Conboy et al., 2020; Wilden et al., 2013).

*Transforming* suggests that the firm will implement the reconfigured business model (Torres et al., 2018), by orchestrating its resources and assets or acquiring new ones in order to move on to the new phase. In other words, at the stage of transforming, the firm needs to implement the necessary steps, such as the orchestration of resources, re-engineer any processes and implement any decision making required that will ensure sustainability (Teece, 2012, 2018).

**Fig. 1:**
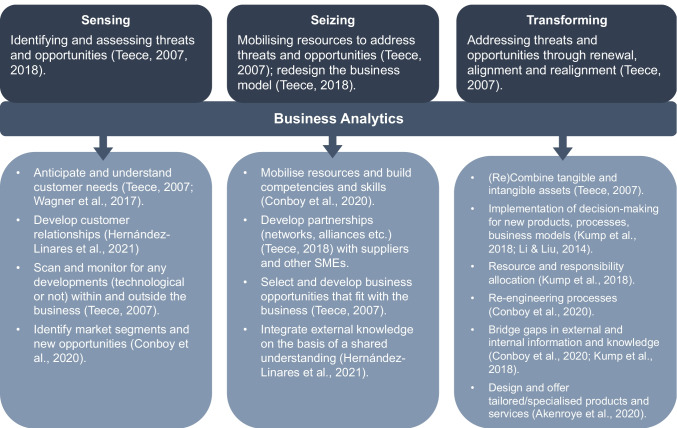
Dynamic Capabilities and activities that can be supported by Business Analytics

Across these dynamic capabilities, whether a firm will be able to respond to external circumstances depends with decision-makers’ interpretation of the crisis (Bullough et al., 2014). Against this background, we argue that BA can play a critical role in supporting decision-makers by helping them make sense of it (Zamani et al., 2021). Existing research shows that BA is valuable for the firm (e.g., Akter et al., 2016; Sharma et al., 2014), being a dynamic capability in and of itself (Torres et al., 2018), or supporting the development of higher-order dynamic capabilities (Ciampi et al., 2021). In addition, BA can support decision-makers during sensing, seizing, and transforming, particularly during the time pressure of exogenous shocks. In Fig. 1, we indicate the activities in which BA can contribute towards achieving each dynamic capability As such, exploring how BA enable business model transformation through the theoretical lens of dynamic capabilities, allows us to explore systematically and disentangle the processes and activities a firm goes through as it considers the BA insights during decision making.

### The Small-Medium Enterprise Context

Within the context of Small-Medium Enterprises (SMEs), business model dynamics can be challenging for two main reasons.

First, the usual tools, e.g., business model canvas, foresight cards and web-based tools, such as the e3value modelling tool^1^ (Athanasopoulou & De Reuver, 2020) that support business model dynamics are typically designed with larger businesses in mind; thus, they do not account for SMEs’ limited resources (Miller et al., 2020), the particularities of SMEs’ organisational features or their lack of strategic capabilities (Cosenz & Bivona, 2020). Therefore, SMEs often adapt and reinvent business models using tailor-made approaches that focus on their own idiosyncrasies (Bianchi et al., 2018). Second, digital technologies, such as BA, which can be invaluable for a firm’s business model (Haaker et al., 2021; Pateli & Giaglis, 2005), require investments in IT infrastructure and IT capabilities; as such, for an SME with limited resources (Ho et al., 2019), the implementation and use of BA can be challenging.

Having said that, for start-ups in particular, a specific type of an SME, business model dynamics seems to be an easier endeavour. Start-ups are characterised by innovation and creativity that enhance the transformation mentality, which is not often found in mature or well-established firms (Braun et al., 2018). Start-ups tend to have an entrepreneurial spirit that facilitates shorter and agile trial-and-error cycles in how they transform their business model contrary to incumbent firms, which more often than not have an established positioning and re-engineer their resources and processes less frequently (Leih et al., 2015). Along these lines, several start-ups explicitly leverage the more innovative and often still emerging technologies to design, adapt and innovate their BMs (Hartmann et al., 2016).

Start-ups tend to underutilise and exploit analytics as means to better understand their customer needs and offer relevant services (Behl et al., 2019), e.g., to design customer acquisition and retention strategies (Sayyed-Alikhani et al., 2021), for product development purposes (Berg et al., 2018), and more rarely to improve their internal process e.g., to prioritise projects (Zamani et al., 2021). Even in these cases in which BA might be underutilised, BA introduce an important opportunity for start-ups and SMEs more generally (Sheng et al., 2020; van Rijmenam et al., 2019). Smaller firms are closer to their clients and have low-level knowledge regarding their collaborations and partnerships, which, when combined with BA insights, can increase their overall competitiveness despite their limited resources (O’Connor & Kelly, 2017). It is evident that the lack of recourses and capabilities leads smaller businesses in leveraging BA usually for customer purposes, however the use of BA to improve internal processes and alter their business models, has been largely understudied. Such an endeavour of course requires appropriate BA implementations, and careful integration of BA within existing organisational processes to enhance the practice (Wang & Wang, 2020). Often, such symbiotic relationships are observed within start-up environments, whereby business models and organisational processes are frequently in and of themselves data-driven (Hartmann et al., 2016).

To date, however, little empirical research exists explaining how exactly BA may support the adaptation or innovation of the business model, especially within the context of SMEs. While not specifically for the SME context, a recent study has confirmed empirically the positive relationship between BMI and analytics, indicating that analytics support the firm’s entrepreneurial orientation (Ciampi et al., 2021). Indeed, we can posit that BA can maximise the chances of successfully adapting and innovating the business model and allow the firm to move to a more viable position when the endeavour is informed and driven by data. Equally, however, we posit that BA in and of itself is not enough. We argue that a firm will need to exhibit capabilities (e.g., absorptive capacity, dynamic capabilities), which will support the firm in exploiting BA insights by making sense of the evolving environment and competition, making data-driven decisions and being ready and able to change (Griva et al., 2021b; Mikalef et al., 2017).

## Methodology and Case Description

In this paper, we present an interpretive single case study (Dyer & Wilkins, 1991; Walsham, 1995) of a Greek start-up, called TrackApp,^2^ where we have been consulting for the past three years. While in this paper we are focussed on how TrackApp has responded to the exogenous shock as far as its BM is concerned, our consulting work has provided us with unlimited access to staff, data and documents and deep knowledge regarding their activities and the surrounding context.

We chose to build on a single case study because of the design’s suitability for our research question. A single case study can provide rich insights without decoupling these from the contextual particularities of the studied phenomenon (Shepherd & Suddaby, 2017). We purposefully chose TrackApp as an exemplar case because it offers a unique opportunity to explore the phenomenon of BM dynamics during a crisis, while letting us investigate how the start-up moved from BMA to BMI. The start-up leverages BA to inform and shape its internal processes, and to develop its products and services for its clients, i.e., its entire business model is data-driven and BA-enabled. In addition, our long-term relationship with TrackApp and our deep immersion into the ‘field’ allowed us to follow a more naturalistic approach, whereby we were able to act as quasi-insiders (Westney & Van Maanen, 2011).

### Research Site

TrackApp was founded in 2018, however, the two founders (R1 and R2) have been working on their idea as early as 2016, while they were doctoral students. Following their graduation, R3 joined their team and they started building and expanding their idea, taking advantage of their multidisciplinary studies and complementary backgrounds. In 2018 they launched TrackApp as a start-up. Since then, the team has expanded to twelve members overall (Table 1), to include seven employees, one contractor and one external business development manager assigned by the Venture Capital fund that has funded the team.

**Table 1:** Basic information on informants

ID*	Job title	Position	(Formal) Interviews
R1	CEO and General Director	Co-founder	Yes: 2 interviews (1 h each approx.), online chat (follow up)
R2	Head of BA	Co-founder	Yes: 2 interviews (1 h the first, 30′ the second approx.), online chat (follow up)
R3	Tech lead/IOS Developer	Co-founder	Yes: 1 interview (1.5 h approx.)
R4	Marketing & Product Manager	Employee	Yes: 1 interview (1 h approx.)
R5	Content Manager	Employee	Yes: 1 interview (30′ approx..)
R6	Machine Learning Expert	Employee	Yes: 1 interview (1 h approx.)
R7	Data scientist 1	Employee	Yes: 1 interview (1 h approx.)
R8	Data scientist 2	Employee	Yes: 1 interview (1 h approx.)
R9	Operations manager	Employee	No
R10	Android developer	Employee	No
R11	Product design	Employee	No
R12	Business Development Manager	Venture Capital fund	Yes: 1 interview (1.5 h approx.)
R13	Backend developer	Contractor	No

*All names replaced with a code for confidentiality purposes

TrackApp builds on the concept of proximity and location-based services. The team has developed a platform that exploits Global Positioning System technology (GPS) and monitors users’ movement within the city. End-users use the TrackApp mobile application on their smartphones to discover points-of-interest around the city, earn points and redeem instant rewards (e.g., vouchers, discounts, samples). TrackApp has a number of B2B customers (both retailers and suppliers), whom they provide with analytics reports and insights via their BA platform. Via the TrackApp platform, retailers and suppliers can monitor insights about the number of visits by TrackApp users, the number of rewards unlocked, customer flows, user personas, competition insights etc.

### Covid-19: The Exogenous Shock

In February 2020, Greece started having the first confirmed Covid-19 cases. When the pandemic first hit Greece in February 2020, TrackApp’s original business model was built on a reward scheme, whereby end users were being incentivised to visit as many locations as possible to unlock rewards and receive credit and coupons for retail stores. In mid-March 2020, the Greek government announced the first restrictions, requesting the closing down of shopping centres, restaurants, and other retail shops throughout the country.

However, being a data-driven start-up, TrackApp had been monitoring the uptake and utilisation of their services and they were thus able to detect early on and ahead of the government announcement that their external environment and the market were rapidly changing. They were able to sense that the soon-to-be announced social distancing measures and restrictions would result in negative impacts on their profitability. In the long term, failure to adapt would have almost certainly threatened TrackApp’s survival.

### Data Collection

**Fig. 2:**
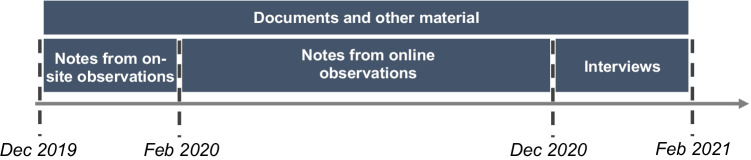
Data gathering during our interaction with the start-up

The primary source of empirical data relates to naturally occurring material (Cunha et al., 2019), collected via multiple means and sources (Table 2), in line with the case study research guidelines (Yin, 2003) and ethnography’s principles (Denzin, 1996). Our observations span a period of 12 months, during which we had online meetings with the members of the start-up and one-to-one meetings primarily with the three founders. Most meetings were focussed on daily operations, but at least five meetings were of strategic importance, where the focus was on discussing the impacts of the social distancing restrictions on the business model, insights from the business analytics platform and the adaptation of the business model, which allowed understanding the overall process. These observations were complemented with empirical material collected via semi-structured interviews with TrackApp members, including follow up interviews and online chats (Table 2). Figure 2 provides the timeline of data collection. The interviews were centred around the core research question and were guided by a set of interview questions, which were used only for probing purposes. The informants were asked to discuss how they have been experiencing the impact of the Covid-19 pandemic individually, as a team and as a business. Also, they were asked regarding the use of business analytics and the insights they have been receiving from their platform, as well as the decisions that have led up to the adaptation of the business model and the actual steps they had taken. For example, they shared:

- what was the role and how they leveraged BA in their processes;
- whether and how the value proposition of TrackApp changed as a result of the process;
- what were the main changes in how they now create and deliver value;
- why and how they made these changes;
- how they organised their processes in order to achieve these changes.

**Table 2:** Empirical material

Observations	Documents and other material	Interviews
• Handwritten notes from observations (post-visit reflections): ○ Prior to Feb 2020: weekly visits (contextual information). ○ Feb – Dec 2020: online meetings, weekly one-to-one meetings (due to social distancing restrictions) on premises.	• Promotional material (e.g., founders’ interviews in the press, YouTube promotional videos). • Briefing and debriefing documents for existing and prospective retail clients. • External communications with customers. • Internal team communications from e-mails, Trello boards, slack channels.	• 11 formal semi-structured interviews: ○ 11.5 h approx. in total. ○ 9 informants interviewed, of whom 2 were interviewed twice and chatted with online for follow-up to clarify and confirm interpretations.

All interviews were conducted in Greek, audio recorded and later transcribed in NVivo for coding purposes. Contextual information was provided through company documents and other material (e.g., press interviews), including through the use of previous observations and experience with TrackApp spanning the preceding three years.

### Data Analysis

We analysed our data following a narrative analysis approach. Narrative analysis is a useful approach when actors share stories with others in an attempt to make sense of events or when they share their experiences with others (Creswell, 2007). It supports the construction of a coherent story during turbulent times out of events and descriptions which may otherwise seem incoherent or fragmented (Frost, 2009). Narayanan et al. (2009) argue for narrative’s added value in investigating episodes of organisational change and processes that pertain of dynamic capabilities. From our point of view, the narrative analysis helps us unpack our informants’ temporal-based accounts into their constituting elements, i.e., the actors, the major and minor events, the contributing factors, and the necessary triggers, which helps us explore, illustrate and add coherence to the processes relating to the business model dynamics at TrackApp.

For the analysis, we followed Narayanan et al.’s approach (2009). In the first phase, we reviewed our observation-based notes and created a core narrative of the business model dynamics at TrackApp. Next, the two authors independently created narratives from the interview transcripts, identifying the key stages of sensing, seizing, and transforming, following from the Dynamic Capabilities theoretical lens, and which were then compared to identify any contradictions and conflicts between interpretations. This process did not result to any major differences, and any minor differences were discussed and resolved on the basis of consultation with the third author, a technique often used in qualitative studies for establishing consistency (e.g., Thomas & Harden, 2008). After achieving consensus, the coding procedure resumed.

The focus was on key decisions made over time and the rationale for each in order to construct a coherent narrative and arrive to what Creswell calls ‘restorying’ (Creswell, 2007). In the third and final phase, we paid attention to identifying the role of Business Analytics in the formation of the key decisions and its function towards sensing, seizing and transforming. Major and minor events taking place within (e.g., new employees, team dynamics) and outside (e.g., lockdown events, government measures) TrackApp were isolated in order to understand the challenges faced by the founders and the rest of the team.

## Findings

**Fig. 3:**
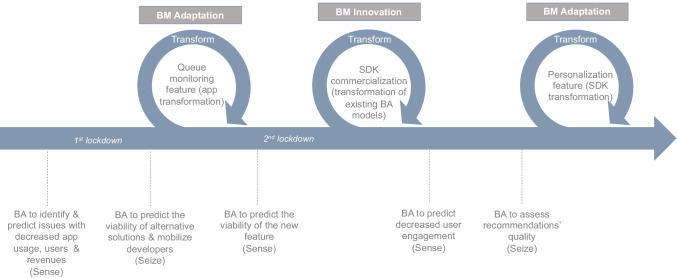
Rounds of BA-enabled Business Model Transformation

Following the Covid-19 exogenous shock, the start-up transformed its business model three times. Each transformation resulted in either a business model adaptation or innovation. Below we present our findings focusing on each business model transformation round (see Fig. 3).

### First Round of Business Model Transformation

Before the announcement of the first lockdown restrictions, TrackApp leveraged BA to identify a drop in active users.

“Our analytics team detected a pattern in decreased footfall rates for spots that used to be crowded […], they also detected that users from the largest city in Greece began underutilising, or even uninstalling our app. Given these, our predictions were discouraging.” (R2)

“We could see what was about to happen… using analytics we were able to identify patterns in the data and make predictions. So, we already knew Covid-19 would have a severe impact on our services.” (R1)

Using sentiment analytics, they also identified a sentiment of fear in people about moving in the city.

“We identified a fear in our followers’ sentiment in social media.” (R4)

Given these insights, they predicted an exponential increase in users uninstalling and not using the app, and thus a loss in their monthly recurring revenues (MRR), even before the implementation of the first lockdown measures.

In addition, the drop in footfall meant that there was less data coming in to TrackApp’s platform. Therefore, TrackApp began experiencing an overall decrease in monthly recurring revenues (MRR) from retailers and suppliers, because, as a result of their business model, the start-up had less and less to offer B2B clients:

“After the first lockdown, indeed, we noticed an overall decrease in our KPIs, MRR, number of active users, everything was collapsing. Without exploiting analytics, we wouldn’t have made correct projections.” (R12)

As tracking and incentivising end users in outdoor environments was no longer possible due to the restrictions, the CEO gathered the team for a brainstorm meeting (online), where all options were open: from thinking around the product as a whole to discussing around new market segments, as well as ‘new ways of doing things’. The only limitation was that proposals and ideas should be in line with the start-up’s values. In the first instance, the discussion was centred around identifying ways to maintain or bring in new end users into the platform. However, considering Covid-19’s high infection rate, this idea was quickly abandoned as TrackApp deemed unethical to incentivise users to circulate more and potentially disobey the non-essential movement restrictions by offering them rewards to do so:

“We could reward users for visiting parks and other outdoor open areas, but we considered this to be an unethical marketing strategy, given the lockdown measures.” (R4)

As a result, the three founders realised they would have to change their product in order to retain their end users and B2B clients. Some of their B2B clientele, such as grocery retail stores, were amongst those who remained open during the lockdown. During one of their meetings, the team noticed that, due to social distancing measures, the operation of grocery stores had changed dramatically since the beginning of the lockdown, as retailers had to control the number of customers entering their stores, leading to long queues.

“We started brainstorming on how we could pivot, to raise awareness and stop losing our users.” (Ada)

“We had a daily stand-up with the more senior team members for brainstorming purposes, various ideas were suggested, for example, a queue monitoring feature, market research surveys to serve our B2B customers and give point to our users, lucky lotteries, these sort of things.” (R1)

Considering their strengths and weaknesses, and that their app was already geo-location enabled, the team decided that a potential alternative to avoid user churn would be to develop a queue monitoring feature for retail shops, such as supermarkets. At the same time, this would aid them in supporting their existing clients’ effort to implement social distancing on premises.

The Tech team who had to develop the queue monitoring feature wasn’t convinced. TrackApp decided to leverage historic data and public datasets to predict and assess the economic viability of the few alternatives they had. Using analytics, TrackApp managed to convince its developers that the queue monitoring feature was a good option, because, by comparison, their predictions for alternatives were discouraging:

“We analysed the number of users that each existing feature brought in the past. We examined user acquisition costs, churn rates. We generated alternative prediction scenarios […]. However, the predictions were discouraging.” (R7)

The start-up developed the queue monitoring feature which was providing live analytics to end-users on the number of customers queuing at each grocery store and a prediction for the timeslot that each store was less crowded. They used the same feature to provide their B2B clients with selling analytics reports regarding consumer traffic, which could be used to adjust their staff requirements. In other words, TrackApp achieved business model adaptation by incorporating BA as a capability in their existing application and product for their B2B customers.

“After a quick and intensive development cycle of ten days, we managed to launch the queue monitoring feature.” (R3)

“The BA team had been working in parallel with the devs to adapt the BA solution and incorporate queue monitoring reports for our B2B clients.” (R2)

### Second Round of Business Model Transformation

Descriptive analytics indicated that, because of the queue monitoring feature, TrackApp stopped losing end-users, acquired some new users, and gained publicity (e.g., interview, social media followers). During the second lockdown that was imposed in November 2020, the feature also attracted the attention of some new clients from the grocery retail sector. However, comparing the performance of older features to this one, the predictions on the long-term viability of the new feature were not optimistic. Although, active users increased, the attraction rate and the respective BA-enabled predictions were discouraging. The same applied for customer engagement. Similarly, despite that Monthly Recurring Revenues (MRR) were slightly increasing, the BA team’s projections indicated short-term revenue and cashflow issues. Overall, the team concluded that the queue monitoring feature in and of itself would not prove enough for the profitability and the sustainability of the start-up.

“Initially it seemed that we had overcome the shock. We retained and regained some users, so we were performing well from an end-user perspective […]. However, we couldn’t achieve profitability.” (R4)

“Looking at the customer-related metrics, the team seemed to perform really good, but they couldn’t convert these metrics into economic value.” (R12)

In light of the BA results, TrackApp started brainstorming to identify further BA-enabled services to bring value to their B2B clients. A promising solution was that of supporting their retail clients to monitor the number of visitors in stores and visualise in-store flows to reduce and avoid floor congestion. This was considered as especially useful for those stores with more than one floors because the imposed Covid-19 restrictions and regulations entailed that retailers were expected to monitor and manage traffic per floor. However, to achieve this, the team would need to adapt their platform to operate within indoor environments. As the Tech Lead underlined during the meeting, this was easier said than done even though their platform was already exploiting location-based data such as data derived by GPS. As such, they decided to exploit location data from IoT technologies, too, such as Wi-Fi antennas and Bluetooth Low Energy (BLE) beacons. The retailer had already installed Wi-Fi antennas, therefore they only would have to install only BLE beacons in the first instance, after securing permission by the store owners.

“This time we considered implementing something ground-breaking, but we had to put a rush in it. We had to move into development fast and roll out the feature as soon as possible because otherwise we would be cashflow negative, and non-viable.” (R1)

“My team had to develop many functionalities to expand our application and our platform. However, I understood that at that time I couldn’t ask them to further assess the potential solution because it would delay the launch date.” (R3)

To entice their B2B clients, TrackApp decided that an attractive offer with added value would be the commercialisation of their BA-enabled location-tracking SDK (Software Development Kit). This would essentially offer a white label app (i.e., opening their SDK to retailers’ existing mobile apps), which would allow customisations for each retailer. From then on, TrackApp adjusted their machine learning (ML) algorithms and accuracy models, from outdoor to indoor locations so that retailers could verify the exact location of their customers in order to use location-based services (e.g., location-based promotions, content, etc.) and produce live BA insights e.g., area traffic, in and out of store queues, heatmaps etc. To further tempt their existing and prospective clients, they anchored their pricing model on the opened sessions from within their SDK. In other words, TrackApp would be paid if, when and as much as retailers would be using the start-up’s application. To accurately monitor their revenues, the company enabled real-time BA to track the number of sessions per client. At the same time, this way they managed to monitor in real-time the performance of this new service (e.g., failures, accuracy reports, service usage, signal attenuations etc.).

“Re-engineering our outdoor location tracking machine learning models to work for indoor environment was a challenge for us. I had to think about new techniques, such as fingerprinting to make our models work.” (R6)

“That was the second time within a single month that we had to add new BA functionalities on our BA platform, heatmaps, shopper flows, and shopping patterns were our ‘coolest’ additions.” (R8)

As a result, in this case. BA worked as an enabler of business model innovation. The team used BA to re-engineer their SDK by adjusting the machine learning models for location tracking. At the same time, once again BA was used to develop a new service for the company’s existing BA platform, offering new insights for retailers. In this round BA was also used as a monitoring tool for real-time evaluation of the performance of the service. This way the company was able to monitor their revenues, and to quickly identify areas that required further re-engineering e.g., retailers’ stores with low location accuracy that required additional fingerprinted methods to avoid signal attenuations etc.

### Third Round of Business Model Transformation

Since launching their indoor offering, TrackApp were monitoring their self-service BA solution and, specifically the number of opened sessions per retailer within the SDK, and the duration of sessions. Based on the BA insights, they realised that although this product seemed to attract end-users and at the same time to produce valuable BA insights for the retailers, after a short period, user engagement started decreasing again, and for the second time the predictions were discouraging. In the short term, the shortfall in user sessions would have a significant impact on their revenues. This meant that another intervention was needed:

“After three weeks we observed that user engagement with the app started decreasing. Then, not unexpectedly, the volume of opened sessions started decreasing, too, and that basically signalled to us that our revenues would soon start decreasing as a result. Long story short, we had to come up with an alternative solution to offer to our clients to keep them interested and keep them engaged.” (R1)

By now, the start-up had already gathered a lot of insights from their BA platform, from the outdoor and the newly developed indoor BA models. What was needed, they envisaged, was a product that would keep engagement high, while respecting the restrictions in place. What the next step entailed was in essence reimagining their offering. They realised that another way of seeing their offering could come from decoupling their reasoning from a data stream approach and from moving away comparing and contrasting the ‘outdoors’ versus the ‘indoors’. Instead, the problem could be framed as a matter of different market segments: from end-users looking for rewards and vouchers while wondering around the city, to those actually entering the stores and looking for similar rewards and vouchers while shopping inside.

“This basically unlocked our way of thinking. We started thinking what sort of new things we could do, we started adding new BA-enabled features to enhance the functionalities of our SDK offering.” (R8)

The underlying goal was to offer in-store personalized promotions and rewards to engage with the existing and new market segments they were now ready to target. As such, TrackApp focussed on exploiting existing resources and competences, its BA models, previously consuming only outdoor tracking data. To carefully design a personalisation solution, the team first explored and assessed the quality of the recommendations exploiting the datasets they had already gathered, i.e., outdoor and indoor location data.

“First, we used analytics to perform segmentation and identify users with similar outdoor behaviours. Then, we exploited the additional data we gathered from the indoor environments. However, we couldn’t really accept the results because the recommendation models were performing very poorly.” (R6)

Due to the recommendation models performing poorly, the team started exploring and applying analytics methods on new data sources that would allow them to increase the quality of their recommendations regarding market segmentation by generating new models. Indeed, with permission, TrackApp accessed retailer’s point-of sales data and loyalty data in order to retrain their models:

“We analysed the sales and loyalty data and combined them with our internal datasets (i.e., indoor and outdoor), this way we managed to improve the quality of the recommendations” (R7)

The third round has led to a more focussed and controlled adaptation, where the business model and the BA platform itself were both refined. Using mining techniques and prescriptive BA capabilities, TrackApp balanced their exploitation and exploration efforts, increased their end-user base, and progressively increased user engagement with their product. With regard to their retail customers, the evidence suggests that, the adaptations made led to added business value, and following the successful roll out, their solution attracted the attention of other retailers, including that of investors.

“We developed a new personalisation feature via exploiting our and retailers’ data, which was then integrated in our offering. User engagement and satisfaction seemed to be increased after the release” (R1)

In this third and last transformation round, the role of analytics was to be leveraged to expand the existing product offering, to combine internal and external assets (i.e., data), and be exploited to develop an additional functionality in the SDK.

TrackApp itself considers this final round of business model adaptation as successful for the aforementioned reasons, but most importantly because this process allowed them to pivot their product and reach out to new markets, which is essential for start-ups. At the same time, they expanded their team, which was among the founders’ goals. Most importantly, as far as the sustainability and the viability of the start-up is concerned, TrackApp secured a new round of funding.

To summarise, Table 3 illustrates how BA enabled dynamic capabilities to help overcome the exogenous shock. This table also summarizes the role of BA in terms of the data sources that were used per each BM transformation phase, and the type of BA exploited each time (i.e., descriptive, diagnostic, predictive, prescriptive) (Maoz, 2013), following the BA classification of BA introduced by Delen and Zolbanin (2018). Note that Table 3 focuses exclusively on the role of BA, excluding other DC steps and processes that might have taken place, but are not BA-enabled.

**Table 3:** The use of BA during the Dynamic Capabilities Process

BM transformation rounds	Dynamic Capabilities Process			Business Model Transformation	Use of BA	
	*Sensing*	*Seizing*	*Transforming*		*Data sources*	*Types of BA*
First round	• Mining patterns in loosing active users in points of interest that used to attract users (before the 1st lockdown). • Sensed sentiment of fear via social media analytics (before the 1st lockdown). • Prediction of app uninstallations via app analytics (before the 1st lockdown). • Prediction of loss in monthly recurring revenues (MRR) (before the 1st lockdown).	• Using BA data sources to predict viability and to mobilise developers. • Applying BA on historic data and public datasets to predict viability of alternative solutions.	• Development of a BA-enabled queue feature to align with the changing external requirements. • Enhancement of company’s BA platform with BA-enabled insights (e.g., prediction of queue congestion).	Business Model Adaptation (BMA)	• Location data (outdoor environment) • Social media data • Application data • Financial data • Historical data (on past products’ performance)	• Descriptive • Diagnostic • Predictive
Second round	• Mining patterns in slightly increasing of active end users, but future BA-based predictions were discouraging. • Customer engagement metrics were increasing, but predictions showed short-term drop-off. • Monthly recurring revenues (MRR) slightly increasing, but projections indicated cashflow issues. • Comparing to past feature performance, predictions for the viability of the new feature were not optimistic.		• Re-engineering of company’s SDK; adjusting BA-enabled location and accuracy ML models ➔ reengineering processes and reconfiguring capabilities. • Developed a new BA service/platform. • Used BA to monitor the performance of the new solution.	Business Model Innovation (BMI)	• Application data • Location data (indoor environment) • Financial data • Historical data (on past features’ performance)	• Descriptive • Diagnostic • Predictive • Prescriptive
Third round	• Descriptive analytics showed that user engagement was decreasing. • Shortfall in number and duration of sessions per retailer. Short-term BA predictions were discouraging. • Decreasing revenues.	• Applying BA on internal datasets to assess the quality of the ML recommendation model. • Applying BA on new data sources to assess recommendation quality.	• BA-enabled in-store personalised promotions and rewards: combination of internal (indoor and outdoor tracking) and external (sales and loyalty) data streams and ML models. • Enhancement of existing products by incorporating BA recommendation models in their existing SDK solution.	Business Model Adaptation (BMA)	• Location data (indoor and outdoor environment) • Application data • Sales data • Loyalty data • Financial data	• Descriptive • Predictive • Prescriptive

## Propositions

On the basis of our findings, we provide in this section four propositions specifically developed around the nuances of SMEs that demonstrate the applicability and value of BA for the SME context. Before one even considers the time pressure element of the pandemic, the first step in this study was to consider whether analytics can play an effective role on business model transformation for SMEs. This is therefore the basis of Proposition 1 (a to c).

- Proposition 1a: *BA enables SMEs to sense the need for business model transformation*.

Identifying changes in a dynamic environment can a be daunting endeavour, especially when the viability of the business is at stake and decisions need to be made under significant time pressure. As shown in our findings, BA supported TrackApp to assess its current situation via: the use of descriptive analytics to generate reports and understand the status of the market (e.g., growth rates, multiples, competition); the use of data mining to identify trends in data; and the use of predictive analytics to predict and project future company’s metrics (e.g., customers/users, revenues, costs). In more detail, our analysis shows that BA can be exploited to proactively identify and predict patterns in behaviours, which can be coupled with the firm’s low-level knowledge of its customers and ultimately arrive to a superior understanding of threats and opportunities in the environment.

- Proposition 1b: *BA enables SMEs to assess solutions and predict the viability of alternative business transformation scenarios.*

Exploiting analytics, the team managed to mobilise its resources and reconfigure its business model using a grounded-on-the-data approach. For example, in our case, during the first round, TrackApp used BA data sources to convince its developers team regarding the promise of the queue monitoring feature and mobilise them towards taking action (round 1). Similarly, during the third transformation round, TrackApp used BA to assess the quality of its recommendation models (round 3). Lastly, BA allows the SME to assess and evaluate the implementation of decisions in real-time and therefore configure and reconfigure as necessary its assets, resources, and processes in order to avoid failure, and achieve the required alignment within an ever-changing environment.

- Proposition 1c: *BA enables SMEs* to execute their business model transformation.

At the same time, BA itself can play a three-fold role during the transformation phase of an SME. First, BA can be incorporated as a capability in existing solutions, products, services, and applications, as it can happened in our case study when TrackApp enhanced its platform with BA-enabled insights, predicting queue congestion (round). Second, it can be leveraged to create a new BA-enabled solution, particularly in the case of start-ups, such as TrackApp, i.e., data-driven firms. This happened in the case of developing the indoors BA solution (round 2). Third, BA can be viewed as a means for continuous evaluation via monitoring deviations and mismatches from the original plans, as indicated during the business model innovation, when TrackApp used BA to continuously monitor and evaluate in real time the implementation and performance of the new solution (round 2). In other words, BA can be leveraged by itself as a product, it can enhance or re-engineer existing products, and it can be used to evaluate the transforming process. In short, we argue that BA can provide decision-makers with information for the SME’s current and future state, and thus instil (greater) confidence in the decision-making process regarding the nature and the direction of the business model transformation tailored to the idiosyncrasies of the SME.

Following the analysis of the use of analytics, the next step was to evaluate the extent to which they can be used in a high-speed manner under extreme time pressure conditions - in this case represented by the pandemic. This now forms the basis for Proposition 2 (a to c).

- Proposition 2a: *BA enables SMEs to rapidly sense the need for business model transformation under intense time pressure.*

During a crisis, such as the one experienced during Covid-19 or the global financial crisis, an SME cannot afford to properly assess its positioning and then make the necessary steps towards adjusting or innovating its business model based on the result of the said assessment, i.e., idle time can be devastating for the viability of the business even if it is used for assessment and reflection. TrackApp, in our study, leveraged BA to quickly sense the situation before the actual implementation of the lockdown measures, and identify areas in its business model that could prove problematic in the short and medium term (round 1). Similarly, BA assisted TrackApp, in accelerating their sensing capabilities during the next rounds of transformation. In more detail, during these rounds, predictive analytics played the role of an emergency alarm, alerting the start-up to act proactively and drive product transformations. This way, it quickly repositioned itself in a way that would safeguard it from the hurdles ahead.

- Proposition 2b: *BA enables SMEs* to rapidly assess solutions and predict the viability of alternative business transformation scenarios *under intense time pressure*.

During the first transformation round, BA accelerated the assessment of the viability of the alternative solutions. By applying predictive analytics to historical datasets, TrackApp realized quickly that past solutions and features would not be viable to tackle this crisis. As a result, they used these discouraging BA results to convince the developers in developing a new feature (i.e., queue monitoring). Without the usage of BA, the team might have lost time in resolving disagreements and in trying to find other solutions to mobilize the developers. Similarly, during round 3, BA assisted the company in quickly assessing the quality of various recommendation models and adjusting their solution. Without the use of BA, the company could realise the potential failure of these recommendations after their actual implementation. Despite the fact that we have evidence to support this proposition in these two rounds, we must admit that during the second phase of transformation, TrackApp made a decision given the time pressure and the shock to accelerate the transformation process by omitting the seizing phase. Therefore, this round did not provide direct evidence that supports this proposition. However, in the rest rounds, we showed that spending time to use BA insights to mobilise resources, identify opportunities and redesign carefully their business model may have been the wiser choice. Therefore, we decided to include this proposition given the logical, sequential flow between sensing, seizing and transforming, and also that it is likely that other or at least some SMEs would realise benefits of using analytics in the seizing process, particularly under time pressure.

- Proposition 2c: *BA enables SMEs to rapidly execute their business model transformation under intense time pressure.*

Being of an entrepreneurial mindset, TrackApp opted to move directly ahead with the information they had from the sensing stage and innovate by leveraging BA for insights and for developing their products and services, too, i.e., as part of their business model itself. In other words, BA provided the start-up with the necessary support and insights to break free from the inertia and rigidity that often characterises SMEs during similar crises, which halts business model transformation when it is most needed (Osiyevskyy & Dewald, 2018).

We draw attention to the two different types of business model transformation and how these relate to the use of BA under time pressure. In the case of business model adaptation, BA played a superior role, guiding TrackApp throughout the process in making incremental changes in the business model, first by sensing the need to enact changes, then by identifying the means and the ways to mobilise their resources and eventually adapting the business model (rounds 1 and 3). Regarding business model innovation (round 2), BA was less useful in the seizing phase, as this was completely skipped because the start-up did not afford to spend time in using BA for mobilising resources and identifying opportunities.

Comparing BM adaptation and transformation, we can say that during the first BM adaptation phase, using BA the firm managed to respond relatively quickly. Although, they spent time on carefully assessing their alternatives in order not to waste resources and effort, the use of analytics assisted them in accelerating this process. Thus, they managed to quickly adapt their solution, but their end-product was not that ground-breaking. In more detail, although the technical solution was considered ground-breaking by the customers (i.e. retailers) due to its technical difficulty and the additional insights provided, according to the BA-enabled indications (see sensing phase, round two), and according to the feedback that the start-up received by its customers, the end-users perceived the new app feature not that novel. One of the reasons according to the retailers that are in close contact with the end-users, is that they are familiar with similar features and educated by other popular apps that offer information on traffic e.g., car traffic on Google maps. This end-users’ perception regarding the lack of novelty and its effect on the monitored analytics, initiated round 2. However, during round 2, this urgent and unstable situation in which TrackApp omitted analytics during seizing, resulted in an even greater utilization of BA, in which TrackApp used BA to create a new product and disrupt the market, both of which are important elements of business model innovation (Foss & Saebi, 2018).

- Proposition 3: *SMEs should utilise different analytics capabilities (*e.g. *descriptive, diagnostic, predictive and prescriptive analytics) at different phases of business model transformation and in different ways.*

TrackApp exploited different analytics capabilities through business model transformation, ranging from plain descriptive and diagnostic, to predictive and prescriptive capabilities (Chandler et al., 2011), motivated particularly due to being a small business. It is notable that, as shown in Table 3, in order to achieve a superior form of business model transformation i.e., business model innovation, all four types of BA seem to be needed to be used in combination. Moreover, although, we did not find a one to one mapping between BA and the Dynamic Capabilities, we can say that prescriptive analytics, which are considered as the superior form of analytics, were exploited only in the transformation phase, e.g., where TrackApp used BA to accurately identify the position of customers in indoor environments (round 2 - business model innovation), and when they used BA to make personalized recommendations (round 3 - business model adaptation).

Regarding the other types of analytics, they were blended in throughout the DC stages. Although descriptive analytics sometimes were utilized in different DC, they mainly played a crucial in the sensing phase, since they were used to assess the current situation in all the three sensing phases. Data mining or diagnostic analytics were used mainly in the sensing and seizing phases to identify patterns in users’ behaviours (rounds 1 and 2) and to sense sentiment of fear in social media (round 1). Predictive analytics were used multiple times during the whole transformation process e.g., during sensing to predict issues before they happen, during seizing to predict the viability of alternative solutions, during transforming to predict congestion in queues.

“We are data-focussed from start to finish, but we are not a large company and we do not have the luxury to use advanced analytics daily in all our operations, prescriptive, machine learning and forecasting are usually used when we are about to launch a new product or feature and disrupt the market. More plain forms of analytics such as descriptive and data mining are those which are more ‘costless’ and we apply more regularly” (R3)

- Proposition 4: *Depending on resource availability, analytics capabilities should be disseminated across SME staff (rather than a dedicated data science person or team).*

While SMEs are often seen as the backbone of a country’s national economy for social and economic reasons, they often face restrictions due to their size and limited financial resources (Eggers, 2020). TrackApp, as a start-up firm is a particularly small firm, and therefore experiences these challenges, too.

“We have our team, and some data scientists, but we can’t say we have a data science team. Larger businesses have entire departments doing just that. But we are data-driven, and this allows everyone at TrackApp to value and understand data and so, each and every one is able to offer feedback and contribute to the analytics part of the business. And they have done so. We have all been contributing, informing our pivoting throughout this year” (R4)

The start-up under examination does not have either a large data science team, or a dedicated team for exploiting BA to transform their internal processes, as would larger firms do. As the findings show, however, that was not necessary after all. Instead, what is necessary is first and foremost an understanding and appreciation of the importance of the data for the business activities of the firm, and what such data may indicate or imply for the business model in the short and long term. In our case, when it was actually required, the whole firm started acting unofficially like a data science team. The CEO and the Business Development Manager were setting the BA-enabled business questions, and even the Tech Lead played this role. The Data Scientists, together with the Development Team, the Marketing Manager and sometimes the Content Manager, were analysing the data and were building the BA models together. The whole team was involved in how they could effectively design their BA insights solution. Via this internal role swapping, insights from BA can be exploited on a needs’ basis, when a more accurate understanding is required or even when critical decisions need to be made. In the case of TrackApp this was made possible by dynamically assembling a temporary team of crucial employees and stakeholders around the project at hand, and particularly, during the seizing and transforming stages whereby contributions from different areas of the start-up were needed in order to execute the business model transformation.

- Proposition 5: *SMEs may use BA to sense several opportunities for business model transformation but only focus on seizing or transforming a subset of these.*

Existing studies (e.g., Chen & Lin, 2021) on larger firms seem to suggest that there is a one-to-one relationship between the sensing, seizing, and transforming stage in the dynamic capabilities process. In other words, a ‘thing’ is sensed, and then a subsequent capability seizes the opportunities afforded by this ‘thing’ and finally a capability transforms the organisation in response to this. While not explicitly argued, it is implicitly indicated that a larger firm will transform its business activities (and potentially its business model) with the aim to improve its operations and processes across all opportunities identified in the sensing stage, or that it has identified as underperforming or as generally problematic during the seizing stage. Conboy et al. (2020), for example, indicate that a firm may use BA to sense an area within the business that is underperforming or is otherwise problematic, and then deploy its dynamic capabilities in that particular area through seizing and transforming. TrackApp, however, in our study followed a different approach:

“Sure… analytics may ‘tell’ you, you need to change this or adapt that, or, say, indicate the need for an additional service. Or it may point to numerous problems. But TrackApp doesn’t have unlimited resources. So, I think they were smart in that they focussed on which of the alternatives could tackle more than one problem, which one was more value-for-money.” (R8)

For example, during the first round, the start-up, via BA, sensed a number of upcoming changes and threats for its business model, but during seizing and transforming, rather than seeking to address all of them on a one-to-one basis, it followed a higher-level approach by developing a single new feature and enhancing its platform. The reason for this was because all the signals sensed were in fact interrelated. Being a smaller firm, TrackApp has only few products and services. Therefore, all the identified threats and issues related to these few areas, and one action could respond and address several of them simultaneously. In other words, for SMEs, the BA-enabled transformation is likely to work as a funnel where the firm starts with several points for consideration and ultimately identifies few relevant actions for addressing them all.

## Discussion

In times of exogenous shocks such as the Covid-19 pandemic, business model dynamics can be the means for aligning with and responding to ongoing changes in the external environment (Saebi et al., 2017). Recent studies have shown that business model innovation, in particular, facilitates a business’ repositioning toward overcoming the adverse effects of the shock itself (Breier et al., 2021; Kraus et al., 2020). Quite often, an underlying assumption is that businesses will be able to employ their dynamic capabilities during the process of adapting and innovating their business model (Randhawa et al., 2020). At the same time, innovative digital technologies, like Business Analytics, support businesses recover from exogenous shocks by enabling processes and services (Papadopoulos et al., 2020), while in and of themselves may be drivers for business model adaptation and innovation (Pateli & Giaglis, 2005). However, most studies of business model dynamics against the backdrop of the pandemic typically focus on the hospitality industry (Breier et al., 2021; Kraus et al., 2020; Sigala, 2020) and on large organisations (Ritter & Pedersen, 2020).

In this study, we have presented the case of a Greek start-up that went through three rounds of business model dynamics in its effort to respond and address the challenges under the time pressure of the exogenous shock of Covid-19. We have adopted the theoretical lens of Dynamic Capabilities and specifically the view of sensing-seizing-transforming to identify the role of Business Analytics in supporting the business model adaptation endeavours.

The start-up went through three rounds of business model dynamics (Fig. 3): when faced with the first impacts of the exogenous shock, the start-up proceeded more cautiously, adapting their business model. This is not necessarily surprising. Literature suggests that decision-makers, especially those who are risk-averse, may be more sensitive to losses than to gains, and therefore less inclined to proceed with more radical changes (Rissanen et al., 2020). However, when this change proved not enough for the viability of the start-up, the members engaged with a more intensive evaluation of their position and their choices. They shaped a model, that was more ground-breaking in its use of advanced analytics, which they combined with the commercialisation of their Software Development Kit (SDK). This may be considered as an adaptive business model innovation (Foss & Saebi, 2017), because they managed to develop new ways for creating, delivering and capturing value, including updating the entire architecture of its business model to account for these changes. In the final round, it could be argued that the start-up took a step back in order to reflect on the changes and enact focussed refinements rather than radical changes, which suggests a more sophisticated approach to business model adaptation and innovation (Randhawa et al., 2020).

### Implications for Research

The first contribution of our study relates to extending our understanding with regard to the role of Business Analytics for business model adaptation and innovation. Our results indicate that a start-up can leverage descriptive, mining, predictive and prescriptive analytics for addressing and responding to exogenous shocks and renewing its business model. However, the results and impacts of applying each type of analytics are different. In the case of TrackApp, in the first round, the start-up made heavy use of descriptive analytics and data mining to sense the threats and opportunities and, subsequently to predict the outcomes of the potential solutions. In the second round, their efforts were entirely BA-enabled, leveraging predictive analytics, to transform existing machine learning models. Finally, in the third round, to optimise their models for the personalisation features, TrackApp turned to prescriptive analytics. All in all, one could argue that the start-up, from round to round, has been scaling up their use of Business Analytics, where the technology allowed them to move from being cautious, to move to ground-breaking solutions, to refining these further. These findings are largely in line with van Rijmenam et al.’s (2019) metasynthesis. However, we extend and contextualise these to the SME context and specifically to the start-up context, explaining how BA may be used during business model dynamics endeavours, and proving tangible examples for each.

The second contribution of this study relates to how start-ups adapt and innovate in times of crisis. In this study we have highlighted that what is required, on the one hand, is a continuous adaptation and renewal, while on the other hand, to achieve a focussed and successful adaptation, start-ups need to emphasise both exploitation and exploration activities. Combining exploration and exploitation activities has often been considered a balancing act or as a paradoxical phenomenon (Ricciardi et al., 2016); yet being able to do so indicates agility and that the business is able to take advantage of the opportunities in the environment (Sambamurthy et al., 2003). These findings align with studies within innovation-intensive environments where continuous renewal is deemed mandatory for incremental or radical changes (e.g., Andriopoulos & Lewis, 2009; Randhawa et al., 2020). This suggests that the start-up context can be seen as similar to the innovation-intensive one as far as business model dynamics is concerned, including new product development.

A third contribution lies with our analytical explanation regarding BA exploitation by SMEs and the relationship between Business Analytics and Dynamic Capabilities in the SME context. Existing studies show that BA is underutilised (Behl et al., 2019) by SMEs and mainly present successful exploitation of analytics by SMEs merely for customer relation purposes (e.g., Behl et al., 2019; Berg et al., 2018; Sayyed-Alikhani et al., 2021), but not for other purposes such as improvement of their internal processes and transformation of their business models. In this study we offer evidence on how Business Analytics may alert a start-up with regard to threats and opportunities; signpost them to potential opportunities and ways of responding; and help them assess the viability of envisaged BMs. In other words, Business Analytics function as a compass or a roadmap (Zamani et al., 2021) towards recovery, where they enhance the start-up’s dynamic capabilities. However, its’ significance is still high, and our findings show that BA can enhance the dynamic capabilities of the firm, which are critical for business model dynamics. During sensing, within an SME context, BA insights are enrich with the intimate knowledge about clients and products; during seizing which sometimes can be omitted as a phase, BA insights can support the SME to mobilise its resource and make the case for the upcoming changes using a grounded-on-the-data approach and assess the viability of alternative solutions without going through the risk of implementing them; during transforming, BA can be used to evaluate in real-time decisions, and form part of the SME’s products and services, especially for the case of data-driven SMEs, such as start-ups. In other words, BA can support decision-making throughout the entire process of business model dynamics and form part of the final business model. These findings challenge previous current knowledge, which to date has shown as reasonable to expect that sensing needs to be followed up by seizing (Torres et al., 2018).

Arguably, the literature is rich with cases of business model dynamics (e.g., Foss & Saebi, 2017, 2018, 2018; Ricciardi et al., 2016). Most of these highlight that among the main drivers for embarking on such endeavours generally reside externally to the firm and range from stakeholders’ changing requirements (e.g., Casadesus-Masanell & Zhu, 2013) to volatile markets and exogenous shocks (e.g., Bhatti et al., 2021; Breier et al., 2021). This study shows that business model dynamics can support SMEs, and especially start-ups, to respond to such changes, especially because they tend to be more sensitive to resource scarcity and have to operate under greater uncertainty (Ghezzi & Cavallo, 2020). We conclude our study by highlighting that business analytics (descriptive, predictive and prescriptive) can significantly enhance the dynamic capabilities of a start-up in sensing threats and opportunities, and identifying areas where innovation is more warranted and can be more fruitful. It can also be the vehicle through which the actual transformation, i.e., business model dynamics, is delivered.

Our final and over-arching contribution is the identification of a research agenda for SME researchers that can further our understanding of BA for business model dynamics - the combination of which has been overlooked by the existing literature (Ciampi et al., 2021). Future research can build on the five propositions we have put forward to further query into this interesting but complex and not well understood research context. Table 4 presents a research agenda that stems from each the propositions we have developed. Researchers may wish to explore these further to enrich their own, but we consider that the questions contain in this agenda are a useful starting point as they indicate areas for further development.

**Table 4:** A Research agenda for business analytics in SMEs

**Proposition 1a:** *BA enables SMEs to sense the need for business model transformation.*	• How can BA be integrated within BM tools (e.g., canvas) to take into account the needs of an SME? • Which parts of existing SMEs’ BM tools BA affect the most? • How can intuitive judgement be enriched by BA insights in an SME context? • What is the role of BA in each business model transformation phase in SMEs?
***Proposition 1b:*** *BA enables SMEs to assess solutions and predict the viability of alternative business transformation scenarios.*	
**Proposition 1c:** *BA enables SMEs* to execute their business model transformation	
**Proposition 2a:** *BA enables SMEs to rapidly sense the need for business model transformation under intense time pressure.*	• Can BA support SMEs break free from inertia outside the context of time pressure? • What may be the differences between the two contexts? • How can an SME incorporate lessons learned from the process of business model dynamics leveraging BA? • How can we measure the value of the BA-enabled acceleration of SMEs response during crisis? • Can BA accelerate all the phases of the transformation process of SMEs? • Which are the phases that BA act as impediments, and delay the transformation process?
**Proposition 2b:** *BA enables SMEs* to rapidly assess solutions and predict the viability of alternative business transformation scenarios *under intense time pressure*.	
***Proposition 2c:*** *BA enables SMEs to rapidly execute their business model transformation under intense time pressure.*	
**Proposition 3:** *SMEs should utilise different analytics capabilities (*e.g. *descriptive, diagnostic, predictive and prescriptive analytics) at different phases of business model transformation and in different ways.*	• What is the exact role of descriptive, mining, predictive and prescriptive analytics in the transformation process? • Should SMEs treat differently the descriptive, mining, predictive and prescriptive analytics capabilities and how? • Is the use of prescriptive analytics a prerequisite for BA-enabled business model innovation? • Can SMEs achieve business model transformation exploiting more plain analytics capabilities e.g., descriptive analytics?
**Proposition 4:** *Depending on resource availability, analytics capabilities should be disseminated across SME staff (rather than a dedicated data science person or team).*	• How can an SME incorporate BA within its existing tools (e.g., spreadsheets) to support its day-to-day business? • What are the minimum IT skills an SME need to ensure that BA can trigger and enhance its dynamic capabilities? • Which roles should a dynamically assembled/temporary team include for exploiting BA insights (during shocks, under time pressure etc.)? • Which role is more crucial for the success of the team? Is a data scientist a prerequisite of such a team to be formed?
**Proposition 5:** *SMEs may use BA to sense several opportunities for business model transformation but only focus on seizing or transforming a subset of these.*	• What are the factors to consider when choosing where to focus the business model dynamics endeavours, especially when time and resources are limited? • Can BA support such a prioritisation (based on time constraints, risk prioritisation etc.)?

### Implications for Practice

The practical value of this study is stressed when we consider the shareholders of the SME and in particular of the start-up ecosystem. First, BA for SMEs can be invaluable. Despite the fact that SMEs use limited BA capabilities to mainly develop products and understand their customers, this study can inspire and educate small companies on how they can use and benefit from analytics in various ways. This study pinpoints the broader role of BA to support SMEs during times of crisis, to sense upcoming changes in the internal and external environment, to proactively take measures and position the business accordingly for a more sustainable future. BA can drive significant changes in the business models of the SMEs, as, our study shows, the insights deriving from BA can indicate the solution, i.e., the new business model, but at the same time, BA can be part of the solution, especially for those SMEs that are more innovative or data-driven, such as start-ups.

Despite the lack of resources in SMEs, e.g., IT skills and capabilities and financial resources, small businesses should be inspired by exemplar start-ups, and the case presented in this study. They should think out of their box and act as a living organisation which can formulate a BA team when most needed. Through our study, we have shown that, BA can still be indispensable for supporting the firm towards its path to recovery, as leveraging BA can compensate for the lack of such resources by triggering and enhancing the SME’s dynamic capabilities within the firm and towards business model adaptation or innovation. These are significant learnings for SMEs and start-ups. Being aware of such issues, the start-up ecosystem in particular, e.g., incubation and acceleration centres, can be benefitted significantly. Incubators may use these results to build roadmaps and educate the start-ups on how they can be agile, act proactively and fast in their task via exploiting analytics.

Our propositions can be viewed as significant learnings to indicate the difficulties and complexities decision-makers may be faced with under the time pressure of an exogenous shock, where Covid-19 serves merely as an example. Although, our case is particular to the Covid-19 context, the overall crisis resembles the impediments, the difficulties, and the fast-changing environment that start-ups face every day. Thus, being aware of such issues, entrepreneurs may manage to act proactively and use these results for learning purposes to avoid failures in similar situations.

Even though resources and capabilities are always a major concern for SMEs, they can be viewed as paths forward. Given this, SMEs can convert the crisis situation into an opportunity to develop synergies among them or within their supplier and collaborator networks to access IT skills, develop complementarities, and make partnerships (Müller et al., 2018). Although this is not explicitly discussed in the manuscript, inspired by this situation, after the first shocks and after the third transformation round in which the team managed to create a viable model, TrackApp itself started thinking of building an external network of collaborators. Their goal was to be able to maximise the value of BA via leveraging external skills, infrastructure and capabilities on a needs basis for tackling similar future crises.

## Conclusions and Limitations

In this study, we followed an intensive single case study approach and we used observational and interview-based empirical material primarily. There are many ways in which the study could have been executed differently to address our research question. For example, rather than focusing on a single start-up, one could possibly conduct a multiple case study design, with the view to cross-compare responses from multiple start-ups. Equally, an opportunity would be to inquire into start-ups that have been incubed or not and focus on the support provided by the incubator in order to address the changing requirements from the external environment, or similarly, how the incubator supports them in building and enhancing their dynamic capabilities.

Also, intensive case study designs provide researchers with rich empirical material, not all of which will always fall within the primary scope of the research. An example of this in our research is the preliminary evidence regarding the centrality of the start-up’s value during decision-making. While this is an interesting finding in itself, we did not pursue it further because we were solely interested on these to the extent that they trigger next steps. In addition, research suggests that the values of start-ups often are those of their founders, and that with time, they may get adapted to the realities of their environment (Ling et al., 2007), particularly when the start-up is threatened with failure. However, we do consider that future research within the domain of start-up values would be particularly interesting, especially for uncovering the influence of culture on decision making.

Another limitation is the choice of case. In TrackApp, business analytics is ubiquitous: it is a facilitator for operations, a mediator for new ventures, the outcome of their processes and an enabler of the business model itself. Potentially, had we focussed on another start-up, whereby business analytics had a lesser role, our findings would have been different. However, considering that most start-ups are data-driven (Hartmann et al., 2016; Hilbig et al., 2018), we believe that the particular start-up is fairly representative of the start-up scene. On a related note, while the purpose of this study was to develop propositions relevant to SMEs, we have no doubt that at least some propositions may be extremely relevant to organisations in general. Therefore, we suggest that general management and analytics researchers also test these propositions in larger organisational settings.

We also need to highlight that in this study that we do not explore Dynamic Capabilities in full but rather only to the extent that they relate to Business Analytics. We used the theoretical lens of Dynamic Capabilities to probe into how BA support or enable business model dynamics, such as the adaptation and the innovation of the business model, as our findings showed. Our analysis was thus focussed on identifying and unpacking the relationship between analytics and business model dynamics, and on exploring if and how analytics activate or support dynamic capabilities for business model adaptation or innovation. As such, our findings regarding the sensing, seizing and transforming capabilities need to be interpreted from the perspective of business analytics, i.e., those activated by Business Analytics, rather than as being the full spectrum of dynamic capabilities of the start-up. In addition, we acknowledge that Dynamic Capabilities and Business Analytics may be useful conceptual lenses for exploring other relevant areas as well, as for example product development (Hassani & Mosconi, 2022). Indeed, some of our findings indicate that Business Analytics may help inform the design of products and services in light of a firm’s dynamic capabilities. As such, we consider that future research could focus on establishing the role of BA for product development, and how this is perceived by the end-users.

## Footnotes

1 https://www.thevalueengineers.nl/, last accessed 13/11/2021.

2 The name has been redacted for confidentiality purposes.
